# SARS-CoV-2 Infection in Dogs and Cats from Southern Germany and Northern Italy during the First Wave of the COVID-19 Pandemic

**DOI:** 10.3390/v13081453

**Published:** 2021-07-26

**Authors:** Julia Klaus, Eric Zini, Katrin Hartmann, Herman Egberink, Anja Kipar, Michèle Bergmann, Carlo Palizzotto, Shan Zhao, Francesco Rossi, Vittoria Franco, Federico Porporato, Regina Hofmann-Lehmann, Marina L. Meli

**Affiliations:** 1 Clinical Laboratory, Department of Clinical Diagnostics and Services, and Center for Clinical Studies, Vetsuisse Faculty, University of Zurich, Winterthurerstrasse 260, 8057 Zurich, Switzerland; rhofmann@vetclinics.uzh.ch (R.H.-L.); mmeli@vetclinics.uzh.ch (M.L.M.); 2 AniCura Istituto Veterinario Novara, Strada Provinciale 9, 28060 Granozzo con Monticello, Novara, Italy; ezini@vetclinics.uzh.ch (E.Z.); carlo.palizzotto7@gmail.com (C.P.); francesco.rossi@anicura.it (F.R.); vittoriafranco1@gmail.com (V.F.); federico.porporato@anicura.it (F.P.); 3 Clinic for Small Animal Internal Medicine, Vetsuisse Faculty, University of Zurich, Winterthurerstrasse 260, 8057 Zurich, Switzerland; 4 Department of Animal Medicine, Production and Health, University of Padova, Viale dell′Università 16, 35020 Legnaro, Padova, Italy; 5 Centre for Clinical Veterinary Medicine, Clinic of Small Animal Medicine, LMU Munich, 80539 Munich, Germany; hartmann@lmu.de (K.H.); n.bergmann@medizinische-kleintierklinik.de (M.B.); 6 Department of Biomolecular Health Sciences, Faculty of Veterinary Medicine, University of Utrecht, 3584 CL Utrecht, The Netherlands; H.F.Egberink@uu.nl (H.E.); zhaoshan419@hotmail.com (S.Z.); 7 Institute of Veterinary Pathology, Vetsuisse Faculty, University of Zurich, Winterthurerstrasse 268, 8057 Zurich, Switzerland; anja.kipar@uzh.ch; 8 Research Center of Swine Disease, College of Veterinary Medicine, Sichuan Agricultural University, Chengdu 611130, China

**Keywords:** SARS-CoV-2, surveillance, prevalence, domestic animals, RT-qPCR, serology, one health, antibody, zoonosis

## Abstract

Severe acute respiratory syndrome coronavirus 2 (SARS-CoV-2) has affected millions of people globally since its first detection in late 2019. Besides humans, cats and, to some extent, dogs were shown to be susceptible to SARS-CoV-2, highlighting the need for surveillance in a One Health context. Seven veterinary clinics from regions with high incidences of coronavirus disease (COVID-19) were recruited during the early pandemic (March to July 2020) for the screening of patients. A total of 2257 oropharyngeal and nasal swab specimen from 877 dogs and 260 cats (including 18 animals from COVID-19-affected households and 92 animals with signs of respiratory disease) were analyzed for the presence of SARS-CoV-2 RNA using reverse transcriptase real-time polymerase chain reaction (RT-qPCR) targeting the viral envelope (E) and RNA dependent RNA polymerase (RdRp) genes. One oropharyngeal swab from an Italian cat, living in a COVID-19-affected household in Piedmont, tested positive in RT-qPCR (1/260; 0.38%, 95% CI: 0.01–2.1%), and SARS-CoV-2 infection of the animal was serologically confirmed six months later. One oropharyngeal swab from a dog was potentially positive (1/877; 0.1%, 95% CI: 0.002–0.63%), but the result was not confirmed in a reference laboratory. Analyses of convenience sera from 118 animals identified one dog (1/94; 1.1%; 95% CI: 0.02–5.7%) from Lombardy, but no cats (0/24), as positive for anti-SARS-CoV-2 receptor binding domain (RBD) antibodies and neutralizing activity. These findings support the hypothesis that the prevalence of SARS-CoV-2 infection in pet cat and dog populations, and hence, the risk of zoonotic transmission to veterinary staff, was low during the first wave of the pandemic, even in hotspot areas.

## 1. Introduction

Shortly after the emergence of severe acute respiratory syndrome coronavirus 2 (SARS-CoV-2) in humans in late 2019, the susceptibility of animals and the implications of this novel coronavirus at the human–animal interface, i.e., in a One Health context, became a widely-researched topic [1,2,3]. SARS-CoV-2, the cause of coronavirus disease-19 (COVID-19) in humans, has rapidly spread worldwide and was declared a pandemic by the World Health Organization (WHO) in March 2020 [4]. Moreover, at that time, the first reports of sporadic SARS-CoV-2 infection in dogs and cats after suspected human-to-animal transmission [2,5,6] became public.

SARS-CoV-2 is a member of the *Coronaviridae* family, and classified in the betacoronavirus genus, along with other mammalian coronaviruses [7,8]. For host cell binding and entry, the viral spike protein on the membrane surface of SARS-CoV-2 uses a host cell receptor that is widespread among mammals, i.e., the angiotensin converting enzyme 2 receptor (ACE2) [9,10]. Domestic and wild felids, ferrets, Syrian hamsters, mink, gorillas, and macaques have been found to be highly susceptible to SARS-CoV-2, and transmission to cohoused animals was reported in these species [11]. By contrast, dogs were shown to have a lower susceptibility than cats [3,12]; however, in both species, natural SARS-CoV-2 infections have been reported [2,5].

In February 2020, the first cases of COVID-19 were identified in Central Europe, and the number of infections with SARS-CoV-2 in humans started to increase. Northern Italy and southern Germany were regions with high numbers of COVID-19 cases in the early pandemic, with a peak of about 2000 confirmed daily new cases at the beginning of April 2020 in Bavaria [13], and 6000 daily cases at the end of March 2020 in Italy, with the main distribution of cases in the northern Italian regions [14]. Cats and dogs are popular pets in Europe, with more than 106 million cats and 87 million dogs living in European households in 2019 [15]. As these species live in close contact with humans, the surveillance of SARS-CoV-2 infections in cats and dogs became important from a One Health perspective.

Previous studies investigating the prevalence of SARS-CoV-2 RNA at the time of the first wave of the pandemic in swab specimens from cats and dogs did not detect positive cases in Italy (494 animals) [16], Rio de Janeiro (96 animals) [17], or throughout Europe, North America and Asia (4616 samples) [18]. However, studies that focused on animals living in COVID-19-affected households, and thus, potentially exposed to SARS-CoV-2, showed varying results. Two studies did not find evidence of infection by RT-PCR in 21 and 56 cats and dogs, respectively [19,20], while another detected viral RNA in 12% (6/50) to 17.6% (3/17) of cats [6,21] and 1.7% (1/59) of dogs [21].

Serological investigations in cats and dogs have detected anti-SARS-CoV-2 antibodies in higher numbers than viral RNA, implicating a higher prevalence of infection. In particular, in animals from COVID-19-affected households, the anti-SARS-CoV-2 antibody prevalence ranged from 10% to 12.8% in dogs and 4.5% to 43.8% in cats [16,20,21,22]. In contrast, during the first wave of the COVID-19 pandemic, the prevalence of anti-SARS-CoV-2 antibodies and neutralizing activity in cats without information regarding the potential exposure to SARS-CoV-2 was 0.69% (6/920) in Germany [23], 0.76% (1/131) in Croatia [24], 0.4% (2/500) in the Netherlands [25], and 14.7% (15/102) in Wuhan [26]. In dogs, the prevalence of neutralizing antibodies was as low as 0.31% (2/654) in Croatia [24], 0.2% (1/500) in the Netherlands [25], and 3.3% (15/451) in Italy [16].

The study described herein was initiated at the onset of the pandemic in Europe and aimed to investigate the prevalence of SARS-CoV-2 infection based on the detection of viral RNA in swabs collected from cats and dogs independent of the history of contact with COVID-19-affected humans and clinical signs. The sampling was conducted in seven veterinary clinics in northern Italy and southern Germany, which were hotspot areas at the time, from March to July 2020. The remains of sera collected from 118 cats and dogs at the same clinics and within the same timespan were additionally analyzed for anti-SARS-CoV-2 antibodies. Moreover, the remains of bronchioalveolar lavage (BAL) and lung tissue samples collected for diagnostic purposes from three dogs, which presented severe respiratory illnesses, were available and analyzed by RT-qPCR and immunohistochemistry for viral antigens, respectively.

## 2. Materials and Methods

### 2.1. Animals and Sample Collection

Samples were collected at seven veterinary clinics from southern Germany and northern Italy, located in Munich, Novara, Bergamo, Lodi, Bologna, Varese, and Florence between March and July 2020. The participating clinics were provided with collection kits, consisting of cotton swabs with plastic shafts (Lidl, Weinfelden, Switzerland and Heinz Herenz, Hamburg, Germany), cytobrushes (Deltalab S.L., Barcelona, Spain), and previously labeled 1.5 mL screw cap tubes (Sarstedt AG & Co. KG, Nümbrecht, Germany) prefilled with 300 µL of DNA/RNA shield solution [27] (Zymo Research Europe GmbH, Freiburg, Germany). Cotton swabs and cytobrushes were used alternatively, based on the veterinarians’ preference. Moreover, protocols with detailed instructions for sample collection, informed consent sheets, storage and shipment information, and an import permit were made available for each of the clinics. The study coordinators in each country obtained approval from the local authorities. In Switzerland, the sample collection was officially approved by the ethics committee of the canton of Zurich (BASEC number 2020–00979) and by the veterinary office of the canton of Zurich (ZH062/20). In Italy, the approval was granted by the AniCura Scientific Council (Stockholm, 6 June 2020). In Munich, the study received approval by the ethics committee of the Ludwig-Maximilian University (no. 208–30−3–2020). Written consent from each owner was collected after they were informed about the study.

Included in the study were dogs and cats which were presented to the collaborating clinics and for whose sampling the owners had given informed written consent. From each animal, oropharyngeal and nasal swabs were collected during the clinical examination. The veterinarians were advised to collect the swabs as close to the oropharynx and as deep in the nasal cavity as was tolerated by the animal. After swabbing the mucosa, the swab tip was placed in the provided collection tube and the overlaying part of the swab was cut with cleaned scissors. The specimens were stored at 4 °C until being shipped in bulk at ambient temperature to the Clinical Laboratory, Vetsuisse Faculty Zurich, Switzerland, for further analysis.

For each animal, data were collected via an online questionnaire (Google Forms, Google LLC, California, United States). The animal’s sex, neutering status, age, health status including the major clinical sign in sick patients, and data on the household (postal code and contact with COVID-19-affected humans) were documented. A total of 2257 swabs including the associated datasets were returned.

Serum samples from 118 cats and dogs, of which 87 were also tested by RT-qPCR, were made available from the clinics in Munich, Bergamo, and Novara. These were leftovers from samples that had been collected for diagnostic purposes unrelated to the study. The sera were stored at 4 °C and shipped at an ambient temperature to the Clinical Laboratory, Switzerland, for serological testing.

From three dogs with severe respiratory disease, the remains of BAL (*n* = 2) and the remains of lung tissue (*n* = 2), which were collected for diagnostic purposes, were submitted. After arrival of the samples at the Clinical Laboratory, the swab, serum, BAL, and tissue samples were stored at −80 °C or analyzed immediately.

### 2.2. Molecular Analysis

Prior to nucleic acid extraction, the samples were prepared as previously described [27,28,29,30]. Briefly, the collection tubes containing the swabs (cotton swabs or cytobrushes) immersed in DNA/RNA shield were thawed at 4 °C and resuspended by vortexing and incubation at 42 °C for 10 min. The liquid was retrieved from the swab after centrifugation, as described in [30]. Nucleic acid extraction was performed with 200 µL of sample input volume. A volume of 90 µL of total nucleic acid (TNA) was extracted using a MagNA Pure LC 2.0 instrument (Roche Diagnostics AG, Rotkreuz, Switzerland) with either the MagNA Pure LC Total Nucleic Acid High Performance Kit or the MagNA Pure LC Total Nucleic Acid Kit (Roche Diagnostics AG) according to the manufacturer’s instructions. For each batch of extraction, a negative control (phosphate-buffered saline (PBS) without Ca^2+^ and Mg^2+^, Life Technologies Ltd., Paisley, UK) was included to monitor for cross contamination. The two BAL samples were processed like the swabs, but without the preparation steps before extraction. Due to limited availability of the MagNA Pure LC Total Nucleic Acid High Performance Kit, the MagNA Pure LC Total Nucleic Acid Kit was used for 725 (32%) of the samples. A further 24 samples were extracted manually using the Qiamp viral RNA mini kit (Qiagen Instruments AG, Hombrechtikon, Switzerland) with 140 µL of input sample volume and 60 µL elution volume, according to the manufacturer’s instructions.

RNA was extracted from lung tissue stored in DNA/RNA shield, using the QIAGEN RNeasy mini kit (Qiagen Instruments AG). A total of 30 mg of tissue was resuspended in 700 µL of RLT buffer including beta-hydroxybutyrate and disrupted in a 2 mL Precellys^®^ CK14 tube including 1.4 mm ceramic (zirconium oxide) beads in a Precellys^®^ 24 tissue homogenizer (Bertin Technologies SAS, Montigny-le-Bretonneux, France) for 2 × 30 s at a speed of 5000/min. After homogenization, 650 µL of the fluid was transferred to a QIAshredder spin column (Qiagen Instruments AG) and centrifuged at full speed for 2 min, followed by RNA extraction according to the manufacturer’s instructions. For RNA extraction from the formalin-fixed, paraffin-embedded lung specimen (routine paraffin wax embedded and provided by the Laboratorio di Analisi Veterinarie la Vallonea S.R.L, Passirana di Rho, Italy), two sections (20 μm) were prepared and deparaffinized by incubation with 1 mL xylene and vortexing until the paraffin had resolved. After centrifugation at high speed for 2 min, the supernatant was carefully collected and mixed with 1 mL absolute ethanol by vortexing, followed by centrifugation at high speed for 2 min. After careful removal of the supernatant, the tube was dried with an open lid at 37 °C for approximately 10 min until all residual ethanol had evaporated. The QIAGEN RNeasy FFPE Kit (Qiagen Instruments AG) served for RNA extraction, following the manufacturer’s protocol, and collecting the extracted RNA in 30 μL of RNase-free water.

**Figure 1:**
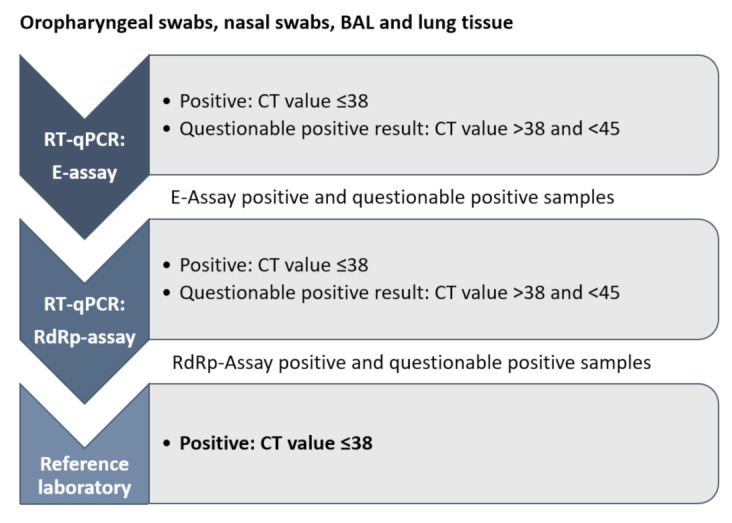
Workflow of molecular analysis applied. The extracted nucleic acids from all oropharyngeal swab samples (*n* = 1132), nasal swabs samples (*n* = 1125), bronchioalveolar lavage (*n* = 2), and lung tissue samples (*n* = 2) were run in the reverse transcriptase real-time polymerase chain reaction (RT-qPCR) targeting the viral envelope (E) gene. Positive and questionable positive samples were further analyzed by RT-qPCR targeting the RNA dependent RNA polymerase (RdRp) gene. If positive or questionable positive after both RT-qPCR assays, the results were confirmed by the reference laboratory of Switzerland, the Swiss Federal Institute of Virology and Immunology (IVI) and stated as positive if the cycle threshold (CT) values resulted ≤38.

For the detection of SARS-CoV-2 RNA, a real-time reverse transcriptase-polymerase chain reaction (RT-qPCR) assay was used to amplify a template on the envelope gene sequence (E-assay). In the case of detection of amplification within 45 cycles in the E-assay, an assay for the RNA dependent-RNA polymerase (RdRp-assay) on the open reading frame-1ab gene (ORF1ab) sequence served to confirm the initial result, as shown in Figure 1. The assays were adapted from previously described methods [31] and conducted with the previously described modifications [30]. Both assays were run on an ABI PRISM 7500 Fast Sequence Detection System (Applied Biosystems, Foster City, CA, USA) using 4 µL of TNA and a TaqMan^®^ Fast Virus 1-Step Master Mix (Applied Biosystems) in a RT-qPCR protocol designed by the Swiss Federal Institute for Virology and Immunology (IVI, Mittelhäusern, Switzerland) [31]. Negative RT-qPCR controls (RNase-DNase-free water, AppliChem, Darmstadt, Germany), a negative extraction control (PBS), and a positive RT-qPCR control (in-vitro transcribed RNA control containing three concatenated sequences of RdRp, E, and nucleocapsid (N) SARS-Cov-2 genes: RNA_Wuhan_RdRp-E-N, provided by the IVI) were added in every run [32].

All samples were run in the E-assay, neat and diluted 1:5 in nuclease-free water to detect possible RT-qPCR inhibition. Samples with a detected amplification in the E-assay were subsequently run in the RdRp-assay for confirmation. The samples were judged as positive when an amplification was detected in both assays (E-assay and RdRp-assay) with CT values ≤38. Samples with CT values >38 and <45 in at least one assay were considered to be questionable positives as long as both assays yielded CT values <45.

The extracted TNA of samples with positive or questionable positive RT-qPCR results was forwarded to the veterinary reference laboratory of Switzerland, the Swiss Federal Institute of Virology and Immunology (IVI, Switzerland), for confirmation [31]. The samples were judged to be positive if all assays were positive with a CT value of ≤38 and questionable positive, if the CT values were >38 to 45.

### 2.3. Histology and Immunohistochemistry

The DNA/RNA shield-stored lung tissue from one dog from Bergamo, remaining after sampling for RNA extraction, was fixed in 10% buffered formalin and embedded in routine paraffin wax. Consecutive sections (3–5 μm) were prepared and stained with hematoxylin-eosin for histological examination and for the detection of SARS-CoV-2 N protein by immunohistochemistry, using the horseradish peroxidase method. Briefly, after deparaffination, sections underwent antigen retrieval in citrate buffer (pH 6.0; Agilent Technologies Schweiz AG, Basel, Switzerland) for 20 min at 98 °C, followed by incubation with the primary antibody (rabbit anti-SARS-CoV nucleocapsid protein; Rockland, 200–402-A50) diluted at 1:3000 in dilution buffer (Agilent Technologies Schweiz AG) overnight at 4 °C. This was followed by blocking of endogenous peroxidase (peroxidase block, Agilent Technologies Schweiz AG) for 10 min at room temperature (RT) and incubation with the secondary antibody, EnVision +/HRP Rabbit (Agilent Technologies Schweiz AG) for 30 min at RT and EnVision FLEX DAB + Chromogen in Substrate buffer (Agilent Technologies Schweiz AG) for 10 min at RT, all in an autostainer (Dako, Agilent Technologies AG). Sections were subsequently counterstained with hematoxylin.

### 2.4. Serological Analysis

#### 2.4.1. Enzyme-Linked Immunosorbent Assay (ELISA)

The sera were tested using an in-house established, enzyme-linked immunosorbent assay (ELISA) for the detection of anti-SARS-CoV-2 spike glycoprotein receptor binding domain (RBD) antibodies, as previously described for cats [30]. Briefly, a 96-well MICROLON^®^, C-bottom, medium binding plate (Greiner-Bio One, St. Gallen, Switzerland) was coated with 200 ng antigen/well using a recombinant spike glycoprotein, RBD SARS-Related Coronavirus 2, Wuhan-Hu-1 with C-Terminal Histidine Tag (NR-52946, BEI Resources, Manassas, USA), by incubating for 3 h at 37 °C and overnight at 4 °C.

Diluted controls and serum samples with a volume of 100 µL/well were pipetted in duplicate on the SARS-CoV-2 Spike RBD-coated plates. The sera were previously heat inactivated at 56 °C for 1 h and diluted at 1:100 followed by incubation at 37 °C for 1 h. Depending on the investigated species, either a goat anticat immunoglobulin G (IgG) horseradish peroxidase (HRP) conjugated secondary antibody (Jackson ImmunoResearch Europe, Ely, UK) or a rabbit anti-dog IgG HRP conjugated secondary antibody (Jackson ImmunoResearch) was added. The conjugates were diluted at 1:3000, and 100 µL/well was used.

**Figure 2:**
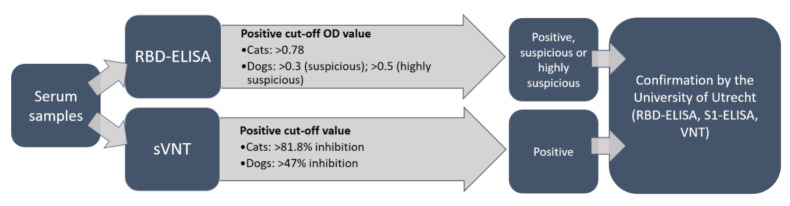
Workflow of serological analysis. The collected sera (*n* = 118) were analyzed in parallel by an enzyme linked immunosorbent assay (ELISA) for the presence of antiSARS-CoV-2 receptor-binding domain (RBD) antibodies and by a surrogate virus neutralization test (sVNT) for neutralizing activity. Positive, suspicious, and highly suspicious samples were confirmed by the University of Utrecht. (OD = optical density; S1 = spike protein subunit 1; VNT = virus neutralization test).

Positive control sera from four SARS-CoV-2 antibody-positive field cats were kindly provided by Dr. Herman Egberink and Dr. Els Broens, Faculty of Veterinary Medicine, University of Utrecht, The Netherlands. As a negative control, a serum sample from a specified pathogen-free (SPF) cat collected in 2017 was used (TVB ZH095/15, [33]). Sera submitted to the diagnostic laboratory from 24 Swiss cats for routine diagnostic purposes (remaining material) between 2 October 2017 and 4 January 2020 [30], and leftovers from sera from 24 dogs (TVB 72/11 and TVB 042/15 [34]) collected in 2013 and 2014, were run as pre-COVID-19 samples. The positive optical density (OD) cut-off value was calculated at six-fold standard deviations above the mean value of reactivity of all serum samples from the pre-COVID-19 cohort for cats [35]. For the dog samples, thresholds to categorize samples as suspicious and highly suspicious were set by the authors. In dogs, a positive control, which was collected as part of a research project investigating animals in COVID-19-affected households in Switzerland (TVB ZH062/20), was added. The control was confirmed as positive by the University of Utrecht. In all samples, a surrogate Virus Neutralization Test (sVNT) was performed, and in samples with positive, suspicious, and highly suspicious results, confirmatory assays were conducted at the University of Utrecht, as shown in Figure 2.

#### 2.4.2. Surrogate Virus Neutralization Test

The commercially available SARS-CoV-2 surrogate Virus Neutralization Test Kit (GenScript Inc., Piscataway, United States) detects antibodies that block the binding of SARS-CoV-2 RBD to the angiotensin-converting enzyme 2 (ACE2) receptor on a cell surface in a species-independent ELISA-like setup, which does not require a biosafety level-3 (BSL-3) laboratory setting. The test assesses the neutralizing activity against SARS-CoV-2 RBD of the spike protein in different species, and was performed according to the manufacturer’s instructions, as also previously described in [30,36].

The OD values were read on a spectrophotometer (SPECTRAmax PLUS 384, Bucher Biotec AG) at 450 nm. Positive and negative controls, provided by the kit, were included in duplicate in every run. The quality control and validation of the results was based on the OD values for positive and negative controls falling in the recommended values. The percentage of inhibition, which is dependent on the titer of anti-SARS-CoV-2 antibodies, was then calculated with the formula (1) below:

Inhibition (%) = (1 − OD value of sample/OD value of negative control) × 100(1)

According to the manufacturer, a result is interpreted as positive for SARS-CoV-2 neutralizing activity when the inhibition was calculated to be ≥20%, while <20% was regarded as a negative result [37]. By including four positive control sera from cats and three positive control sera from dogs, as well as 24 feline and 24 canine pre-COVID-19 samples, positive cut-off values for both species were calculated at six-fold standard deviations above the mean value of reactivity of the pre-COVID-19 cohorts.

#### 2.4.3. Confirmation of Serological Findings

Serum samples with positive, suspicious, or highly suspicious results in either the RBD-ELISA or sVNT were sent to the Virology Division of the Faculty of Veterinary Medicine, University of Utrecht for confirmation. Confirmation was performed by using two ELISA assays (S1 and RBD protein) and a virus neutralization test with a pseudotyped SARS-CoV-2 spike protein, as described previously [25,38].

#### 2.4.4. Assessment of Cross-Reactivity to Feline Coronavirus

To assess potential cross-reactivity to antibodies against the feline coronavirus (FCoV), which is classified as an alphacoronavirus, 24 feline convenience serum samples with a FCoV immunofluorescence (IFA, [39]) titer ≥ 1:25 were tested with the RBD-ELISA.

## 3. Results

### 3.1. Collected Samples and Characteristics

**Figure 3:**
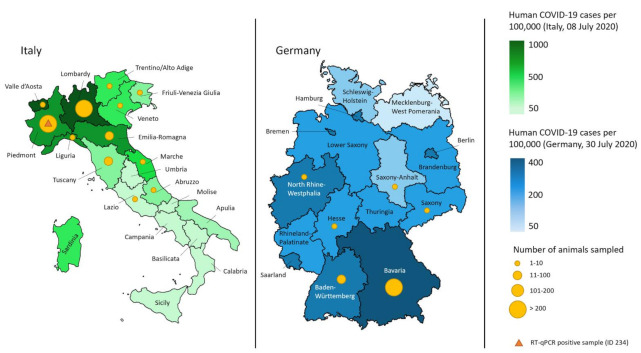
Geographical distribution of swab-sampled animals in comparison to the cumulative human COVID-19 cases per 100,000 residents per region (Italy and Germany). The number of sampled animals per region is shown by the size of the yellow dots, as indicated in the legend. The green and blue color gradient shows the number of cumulative human COVID-19 cases per 100,000. Most RT-qPCR-tested animals resided in Lombardy, Piedmont, and Bavaria. These regions showed the highest cumulative COVID-19 cases per 100,000 in the first wave of the pandemic in these two countries (data from 8 July 2020 and 30 July 2020). The RT-qPCR- positive cat (ID 234) lived in Piedmont (orange triangle); the questionable RT-qPCR positive dog (ID 213) lived in the same area (not indicated). Data source: Robert-Koch-Institute [40], COVID-19 dashboard, Dipartimento della Protezione Civile and ISTAT [14,41].

A total of 2257 swabs from 1137 animals (877 dogs and 260 cats) were included in the molecular analysis. From 255 cats and 865 dogs, both oropharyngeal and nasal swabs were available. From five cats and seven dogs, only oropharyngeal swabs, and from five dogs, only nasal swabs, were submitted for analysis. Most animals lived in Bavaria, Lombardy, and Piedmont. These regions showed the highest incidence of human COVID-19 cases in the first wave of the pandemic (Figure 3).

In 18 cases (12 dogs and 6 cats), potential exposure to SARS-CoV-2 due to living in a COVID-19-affected environment was reported (Table 1). Clinical signs for respiratory disease at the time of sampling was documented in 92 animals (67 dogs and 25 cats), as shown in Table 1.

### 3.2. Molecular Analysis

From the 2257 swabs collected, all nasal swabs (*n* = 1125) tested negative for SARS-CoV-2 RNA. Two of the 1132 oropharyngeal swabs tested questionable positive with CT values ranging from 36 to 43 in the E-and RdRp-assay, while the other 1130 oropharyngeal swabs tested negative for SARS-CoV-2 RNA. A questionable positive result from a cat’s oropharyngeal swab (ID 234) was confirmed as positive by the Swiss reference laboratory (IVI). The other questionable positive oropharyngeal swab, collected from a dog (ID 213), was not confirmed as positive by the reference laboratory. The two BAL and two lung tissue samples, collected from three dogs, also yielded negative results.

Therefore, 0.38% of cats (1/260; 0.38%, 95% CI: 0.01–2.1%) tested positive for SARS-CoV-2 by RT-qPCR, while none of the dogs (0/877) tested positive, but one dog tested questionable positive (1/877; 0.1%, 95% CI: 0.002–0.63%).

### 3.3. Histology and Immunohistochemistry

The histological examination of the lung biopsy from a dog from Bergamo (male, entire, >8 years old) which died from respiratory distress (no necropsy performed) did not show any features indicative of alveolar damage. Changes were restricted to moderate interstitial fibrosis, mild alveolar edema, and mild anthracosis. SARS-CoV-2 N protein expression was not detected in a consecutive section stained by immunohistochemistry.

### 3.4. Serological Analysis

**Figure 4:**
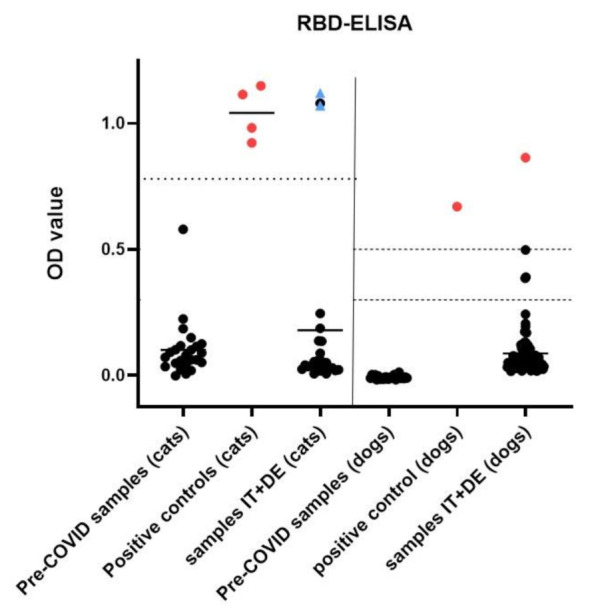
Results of receptor binding domain enzyme-linked immunosorbent assay (RBD-ELISA) on cat sera (left) and dog sera (right). Optical density (OD) values (measured at 415 nm) of cat samples and controls are indicated on the left half, and dog samples and controls are indicated on the right half of the figure. Samples that were confirmed as positive by the surrogate virus neutralization test (sVNT) and the University of Utrecht (S1 ELISA, S1-RBD ELISA, and pseudotyped VNT) are shown in red. The dotted line indicates the positive cut-off value for cats calculated as the six-fold standard deviation above the mean of pre-COVID-19 controls from cat sera (OD value 0.78). A positive cut-off value for dogs was not determined. Instead, samples with an OD value above 0.3 are described as suspicious, and above 0.5 as highly suspicious (dashed lines). One sample from a dog (ID 5) yielded a highly suspicious OD value of 0.86, and was confirmed to be positive by the sVNT and the University of Utrecht. Two sera collected in October and November 2020 from the RT-qPCR-positive cat (ID 234) are indicated as blue triangles (results were confirmed by sVNT and by the University of Utrecht).

**Figure 5:**
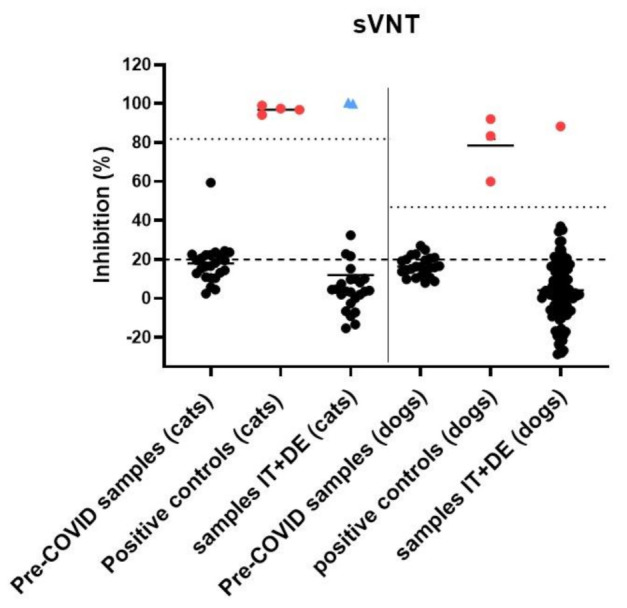
Surrogate virus neutralization test (sVNT) results on cat sera (left) and dog sera (right). The calculated inhibition is indicated as a percentage on the *y*-axis. The dotted lines show the positive cut-off value set for cat and dog samples, by calculating the six-fold standard deviation above the mean of pre-COVID-19 controls inhibition (81.8% in cats and 47% in dogs). The dashed line indicates the positive cut-off set, according to the manufacturer’s instructions, at 20%. Positive samples, which were also confirmed by the University of Utrecht, are shown as red dots. One dog (ID 5) showed neutralizing activity with an inhibition of 88.3%. The two samples collected from the RT-qPCR-positive cat (ID 234) in October 2020 with an inhibition of 99.9% and in November 2020 with an inhibition of 100.5% are indicated with blue triangles.

The sera from 94 dogs and 24 cats collected from March to July 2020 were analyzed for the presence of anti-SARS-CoV-2 spike RBD IgG antibodies (Figure 4) and neutralizing activity (Figure 5). None of the animals had any reported COVID-19 exposure. One dog (1/94; 1.1%; 95% CI: 0.02–5.7%) from Lombardy showed positive results in both serological assays (RBD-ELISA: OD value 0.86 (high suspicion >0.5; sVNT): inhibition 88.3%, (positive cut-off at 47%)) and was confirmed positive at the University of Utrecht by S1 ELISA, S1-RBD ELISA and pseudotyped virus neutralization test (VNT). One of the 24 cats (ID 225) showed reactivity above the positive cut-off value in the RBD-ELISA, with an OD value of 1.08 (positive cut-off at 0.78). However, this result could not be confirmed by either the sVNT or at the University of Utrecht, where the sample was negative in S1 ELISA and pseudotyped VNT, but also yielded positive RBD-ELISA results. The RBD-ELISA and sVNT results obtained at the Clinical Laboratory in Zurich are shown in Figure 4 and Figure 5, respectively.

The sera collected from the RT-qPCR-positive cat (ID 234) in October and November 2020 yielded positive results in the RBD-ELISA, with OD values of 1.07 and 1.12 (positive cut-off value at 0.78), respectively, and in the sVNT, with 99.9% and 100.5% (positive cut-off at 81.8%), respectively. As the samples were not collected within the timeframe set for this study, the results are not included in the prevalence assessment; they are nonetheless included in Figure 4 and Figure 5, indicated by the blue triangles.

### 3.5. Assessment of Potential Cross-Reactivity

To assess potential cross-reactivity to antibodies against the FCoV in the RBD ELISA, 24 feline convenience serum samples with FCoV IFA titers ranging from 1:25 to >1:1600 were tested. Twenty-three FCoV antibody-positive samples (two with a titer of 1:25, three with a titer of 1:100, two with a titer of 1:400, twelve with a titer of 1:1600, and five with at titer >1:1600) yielded negative results in the RBD-ELISA with a mean OD value of 0.26 (range from 0.02 to 0.77). One of the feline serum sample (1/24) with a FCoV titer of 1:1600 showed an OD value of 0.8 (positive cut-off at 0.78). An overall correlation between IFA titer and OD value in RBD-ELISA was not noted.

### 3.6. Description of RT-qPCR or Antibody Positive Animals

The one cat that tested positive in the RT-qPCR (ID 234) subsequently also developed antibodies, as described in a case report recently [42]. This cat was a patient in one of the collaborating clinics in Piedmont in May 2020, presenting with vomitus, diarrhea, tenesmus ani, and weight loss. Three days after collection of the oropharyngeal and nasal swabs, the cat developed respiratory signs, such as coughing, sneezing, and ocular discharge. At that time, the owner experienced COVID-19 like symptoms and SARS-CoV-2 infection was later confirmed in the owner by the detection of anti-SARS-CoV-2 antibodies. The cat was diagnosed with an intestinal B-cell lymphoma and chemotherapeutic treatment was started in June 2020. The cat’s serum samples collected on 14 October and 16 November 2020 yielded positive results in the RBD-ELISA (Figure 4), and neutralizing activity (Figure 5) was detected.

The dog (ID 5) from Lombardy that tested positive in serological assays was male, adult and healthy, and it was not known whether the animal could have been exposed to a COVID-19-affected person. RBD-ELISA and sVNT showed an OD value and an inhibition of 0.86 and 88.3%, respectively. The dog’s oropharyngeal and nasal swab, collected one week after the serum sample, showed negative results in the RT-qPCR.

Although not confirmed as positive, the oropharyngeal sample of one dog (ID 213) from Piedmont showed a low signal, and therefore, a questionable positive result in the two RT-qPCR assays targeting two different genes of SARS-CoV-2. This adult female dog presented with vomiting, but potential exposure to a COVID-19-affected environment was not known at the time of sample and data collection.

## 4. Discussion

In a factsheet published in May 2021, the World Organization for Animal Health (OIE) lists cats as highly susceptible to SARS-CoV-2 infection and dogs as less susceptible than cats, based on the data published to date [43]. However, the epidemiological role of companion animals in the COVID-19 pandemic is not yet fully understood. Dogs and cats are among the most popular companion animal species; they often live in close contact with their owners and can contract SARS-CoV-2 [2,16,21,44]. Experimental studies have shown that cats, unlike dogs, can transmit SARS-CoV-2 to cohoused cats via direct contact or aerosols [3,45,46]. This gives reason to assume that cat-to-human transmission is also a possibility [47]. Proof of virus transmission from SARS-CoV-2 infected pet animals to humans, however, will be difficult to obtain, due to limitations on study design and for ethical reasons [48,49]. Therefore, any studies that shed light on the role of pet animals during the COVID-19 pandemic are of great importance.

In almost all reports of natural SARS-CoV-2 infection in companion animals, human-to-animal transmission was suspected [2,6,49,50,51], and the molecular and antibody prevalence was shown to be higher in cats and dogs living in COVID-19-affected households [6,20,21,22] compared to feral populations or animals with unknown exposure to COVID-19-affected humans [17,26]. Nevertheless, human-to-animal transmission can also occur in unknowingly COVID-19-affected households, e.g., in households with presymptomatic or asymptomatic humans. A meta-analysis which screened 2454 articles that included 663 positive humans, of which 111 were asymptomatic, found the proportion of asymptomatic infections among humans to be as high as 17% (95% CI: 14% to 20%) [52]. Although asymptomatic patients were found to infect slightly fewer contacts than symptomatic patients, the risk of transmission was still significant [53]. Wide-scale monitoring of SARS-CoV-2 infections in companion animals in areas with high incidences among humans during the early phase of the pandemic was the chosen approach in the present study to broadly monitor this novel viral infection from a One Health perspective.

In the present study, dogs and cats were included, which were presented to veterinary clinics regardless of knowledge of potential exposure to SARS-CoV-2. However, the regions where the animals resided were hotspot areas during the first wave of the COVID-19 pandemic, i.e., during the time of sampling. In the study cohort, 18 of the 1137 enrolled animals (1.6%) had reported contact with COVID-19-affected humans and 92 animals (8.1%) showed signs of respiratory disease, which, in humans, would be considered consistent with COVID-19 infection. The fact that only animals that were presented to veterinary clinics were enrolled must be considered when interpreting the results. Indeed, animals undergoing an asymptomatic SARS-CoV-2 infection could potentially be underrepresented in the study population, since mainly diseased animals are presented to veterinary clinics, with few exceptions (76% of animals in this study’s population were reported as sick). Under natural conditions, the periods of potential virus shedding of infected animals might coincide with the time of self-isolation of the owners who might therefore have been unable to present the animals to a veterinarian, missing the early phase of a SARS-CoV-2 infection for sampling. When presented after the owners’ isolation period, the animals might have already overcome the shedding period and developed antibodies against SARS-CoV-2; however, only a small number of serum samples were available for this study. In experimental studies, it was shown that SARS-CoV-2 RNA is cleared within the first 14 days after infection in cats and within 6 days in dogs [3,54]. A study investigating COVID-19-affected households showed RT-qPCR positivity in a cat 32 days, and in two dogs 9 days after the owners’ COVID-19 diagnosis [21]. However, only limited data are available on shedding periods and patterns in companion animals. Our study comprised fewer cats than dogs, which might be explained by a lower representation of cats in the clinics or due to differences in patients’ compliance during the sampling procedure. Nonetheless, our prevalence results can be regarded as representative of the pet cat and dog populations in areas with high COVID-19 incidences among humans during the first wave of the pandemic.

We found the prevalence of SARS-CoV-2 infections to be low among the dogs and cats presented to veterinary clinics during the first wave of the COVID-19 pandemic in hotspot areas. One Italian cat living in a COVID-19-affected household in Piedmont tested positive in RT-qPCR (1/260; 0.38%, 95% CI: 0.01–2.1%). The cat’s oropharyngeal swab sample, which was confirmed as positive by the Swiss reference laboratory, yielded high CT values ranging from 37 to 42, which represent a low viral load. Nonetheless, active infection in this cat was confirmed by the presence of antibodies five and six months after the positive RT-qPCR result. The viral load in this cat was too low for assessment of the viral sequence and comparison with SARS-CoV-2 isolated in humans in Piedmont at the time [42]. None of the 95 German cats tested positive in RT-qPCR. These results are in line with previous studies showing zero prevalence in the detection of viral RNA in Italy (180 samples from cats) [16] and throughout Europe, North America, and Asia (2466 samples from cats) during the early pandemic [18].

None of the 877 dogs (672 from Italy and 205 from Germany) tested positive in RT-qPCR, while only one dog tested questionable positive in our laboratory (1/877; 0.1%, 95% CI: 0.002–0.63%). The dog showed CT values of 36.6 and 43 in both RT-qPCR assays performed at the Clinical Laboratory from the oropharyngeal swab; however, the sample was not confirmed at the Swiss Federal Institute for Virology and Immunology. This discrepancy can be explained by loss of RNA integrity by repeated freeze–thaw cycles and during transport. Additionally, variations between laboratories and the performed assays can affect the results, especially in samples with low viral RNA loads. These low viral RNA loads could be due to the timing of sampling; alternatively, they might represent contamination. This specific dog was presented due to vomiting, but neither vomit, feces, nor subsequent serum were available for analysis. Therefore, SARS-CoV-2 infection of the gastrointestinal tract and possible contamination of the oropharyngeal mucosa due to vomiting cannot be excluded, and the cause of these questionable positive results remains unclear.

The absence of clearly positive RT-qPCR results in dogs in the present study is in line with findings from previous molecular investigations on the prevalence of SARS-CoV-2, where none of the dogs tested positive within cohorts of 314 individuals from Italy and 2150 dogs from Europe, North America, and Asia [16,18]. The results of the molecular detection of the virus are in contrast with those of the serological testing, where one of the 118 canine serum samples analyzed for anti-SARS-CoV-2 RBD antibodies and neutralizing activity (1/94; 1.1%; 95% CI: 0.02–5.7%) was found to be positive. This dog tested negative in RT-qPCR and was not reported to have had exposure to a COVID-19-affected human. Although the route of infection in this case remains unclear, human-to-animal transmission by close contact with infected humans seems likely.

Positive cut-off values for the serological tests were set by including samples from 24 dogs and cats collected before the COVID-19 pandemic as negative controls. In the RBD-ELISA assay, the positive cut-off for cats was set at an OD value of 0.78, which was calculated as the mean OD value of pre-COVID-19 cat samples plus the six-fold standard deviation [30]. The high reactivity in one cat sample (ID 225) could not be confirmed by the surrogate virus neutralization test or by the tests performed at the University of Utrecht, which investigated binding to the S1 protein and virus neutralization. Therefore, the sample was interpreted as negative. However, the RBD-ELISA in Utrecht also yielded a positive result in this specimen. These concordant findings gave reason to further investigate the underlying cause of the high reactivity in the RBD-ELISA. However, the ELISA using SARS-CoV-2 S1 and RBD protein, which were applied by the University of Utrecht, had previously been investigated for cross-reactivity with antibodies against FCoV, and no cross-reactivity was reported [25]. In accordance with our results showing 23/24 FCoV antibody-positive cats having a reactivity below the positive cut-off value in the RBD-ELISA, the positive signal in the sample from cat ID 225 seems unlikely to illustrate cross-reactivity to FCoV antibodies; the FCoV IFA titer of this specimen was 1:400. However, an interference with anti-coronavirus-antibodies cannot be fully excluded. Viruses in the *Coronaviridae* family have demonstrated their ability for interspecies transmission, and recombination events have been illustrated in the past [55,56,57]. An antibody response to a yet-unknown feline coronavirus, although unlikely, needs to be considered. Alternatively, a virus of the genus betacoronavirus present in this cat after transspecies transmission could also be imagined as a possibility for the increased reactivity of this sample in the SARS-CoV-2 RBD-ELISA. Therefore, we recommend that a positive result from a feline serum sample in SARS-CoV-2 RBD-ELISA should be confirmed by at least one other assay, for example, a virus neutralization test.

The positive cut-off for dogs in the RBD-ELISA could not be determined using the pre-COVID-19 cohort sample values and the six-fold standard deviation, as described for the cat samples [30], due to a low reactivity in the pre-COVID-19 samples, which would have resulted in a cut-off value of around zero. Therefore, two cut-off values were set by the authors: samples with an OD value above 0.3 were categorized as suspicious, and samples with an OD value above 0.5 were categorized as highly suspicious. However, all samples were further tested with the sVNT, and samples testing suspicious and highly suspicious in the SARS-CoV-2 RBD-ELISA were sent to the University of Utrecht for confirmatory testing for antibodies binding to SARS-CoV-2 S1 and S1-RBD protein and neutralizing activity. Three samples with suspicious OD values in the RBD-ELISA (OD 0.38–0.5) were confirmed positive by neither sVNT nor by the tests at the University of Utrecht. In these samples, a possible cross-reactivity with antibodies against the canine respiratory coronavirus (CRCoV), another betacoronavirus, and against the canine coronavirus (CCoV, genus Alphacoronavirus), which might also be cross-reactive with the FCoV [58], cannot be excluded. However, the confirmatory tests applied at the University of Utrecht were evaluated for cross-reactivity against CRCoV (positive samples in proxy antigen: bovine coronavirus S1) and CCoV (positive samples in proxy antigen: FCoV type II S1), and no cross-reactivity for the SARS-CoV-2 S1 and RBD protein was reported in those samples [25].

## 5. Conclusions

We found the prevalence of SARS-CoV-2 infection to be low among dogs and cats presented to veterinary clinics during the first wave of the COVID-19 pandemic in hotspot areas. One cat (1/260, 0.38%, 95% CI: 0.01–2.1) was found to be positive for SARS-CoV-2 RNA from an oropharyngeal swab, and was confirmed to have undergone SARS-CoV-2 infection by serology for anti-SARS-CoV-2 antibodies and neutralizing activity. Furthermore, one dog tested positive in the serological assays (1/94, 1.1%). For the verification of RBD-ELISA results, we recommend applying an additional ELISA or VNT, as increased reactivity in the RBD-ELISA may not be specific to SARS-CoV-2 infection.

In conclusion, our findings indicate that cats and dogs did not bear a relevant risk of transmitting SARS-CoV-2 infection in the setting of veterinary examinations during the first wave of the pandemic, even in regions with a high prevalence of infection in people. However, applying proper hygiene measures, as indicated by the Center of Disease Control and Prevention (CDC, [59]), especially when in contact with animals from known COVID-19-affected households, is still advised.

## Figures and Tables

**Table 1:** Signalment, health status, respiratory illnesses and exposure to COVID-19-affected humans of swab sampled animals. Numbers in bold indicate the characteristics of the RT-qPCR-positive cat (ID 234).

Signalment, History, Clinical Signs	Dogs		Cats		Total	
	No.	% of Total Dogs	No.	% of Total Cats	No.	% of Total Animals
**Age**						
<1 year	45	5%	35	13%	80	7%
1–8 years	466	53%	122	47%	588	52%
>8 years	366	42%	**103**	40%	469	41%
**Sex**						
Female	430	49%	**114**	44%	544	48%
Male	447	51%	146	56%	593	52%
**COVID-19 exposure**						
Yes	12	1%	6 *	2%	18	1%
No	145	17%	58	22%	203	18%
Not sure	720	82%	196	76%	916	81%
**Health status**						
Healthy	165	19%	80	31%	245	21%
Sick	688	78%	**174**	67%	862	76%
Not sure	24	3%	6	2%	30	3%
**Respiratory illness ^1^**						
Yes	67	8%	25	10%	92	8%
No	810	92%	**235**	90%	1045	92%

^1^ Includes clinical signs such as coughing and dyspnea, as well as disease processes such as conjunctivitis, stomatitis, rhinitis, pharyngitis, tonsillitis, tracheitis, bronchitis, pneumonia, pleuritis, pleural effusion, pleural edema, and unclassified pneumopathies. * The COVID-19 exposure of the RT-qPCR positive cat (ID 234) was initially documented as “not sure”, but was revised to “yes” after the owner later reported having experienced COVID-19 like symptoms at the time of the cat’s swab sampling and was confirmed to have antibodies against SARS-CoV-2, as reported in detail in a case report [42].

## Data Availability

The data presented in this study are available on request from the corresponding author.
